# Phenolic Compounds as Promising Drug Candidates in Tuberculosis Therapy

**DOI:** 10.3390/molecules24132449

**Published:** 2019-07-04

**Authors:** Muhamad Harith Mazlun, Siti Fatimah Sabran, Maryati Mohamed, Mohd Fadzelly Abu Bakar, Zunoliza Abdullah

**Affiliations:** 1 Department of Technology and Natural Resources, Faculty of Applied Sciences and Technology, Universiti Tun Hussein Onn Malaysia (UTHM), Pagoh Educational Hub, Pagoh 84600, Muar, Johor, Malaysia; 2 Centre of Research for Sustainable Uses of Natural Resources (CoR-SUNR), Universiti Tun Hussein Onn Malaysia (UTHM), Pagoh Educational Hub, Pagoh 84600, Muar, Johor, Malaysia; 3 Natural Products Division, Forest Research Institute Malaysia (FRIM), Kepong 52109, Selangor, Malaysia

**Keywords:** antimycobacterial, phenolic, phytochemicals, tuberculosis drugs, natural products

## Abstract

Tuberculosis (TB), caused by *Mycobacterium tuberculosis* (MTB) remains one of the deadliest, infectious diseases worldwide. The detrimental effects caused by the existing anti-TB drugs to TB patients and the emergence of resistance strains of *M. tuberculosis* has driven efforts from natural products researchers around the globe in discovering novel anti-TB drugs that are more efficacious and with less side effects. There were eleven main review publications that focused on natural products with anti-TB potentials. However, none of them specifically emphasized antimycobacterial phenolic compounds. Thus, the current review’s main objective is to highlight and summarize phenolic compounds found active against mycobacteria from 2000 to 2017. Based on the past studies in the electronic databases, the present review also focuses on several test organisms used in TB researches and their different distinct properties, a few types of in vitro TB bioassay and comparison between their strengths and drawbacks, different methods of extraction, fractionation and isolation, ways of characterizing and identifying isolated compounds and the mechanism of actions of anti-TB phenolic compounds as reported in the literature.

## 1. Introduction

Tuberculosis (TB) is an infectious and fatal pulmonary disease, which has been menacing mankind for millennia and remains a major health concern worldwide [1]. As reported by WHO in the Global Tuberculosis Report 2018, in 2017, the estimated new cases of TB were 10.0 million cases, which was equivalent to 133 cases of 100,000 population. The most estimated number of cases in 2017 occurred in WHO South-East Asia Region, which constituted 44% of total cases reported followed by WHO African Region (25%), WHO Western Pacific Region (18%), WHO Eastern Mediterranean Region (7.7%), the WHO Region of the Americas (2.8%) and the remaining 2.7% by WHO European Region [1]. TB has caused an estimated 1.3 million deaths among HIV-negative people and there were an additional 300,000 deaths from TB among HIV-positive people. Ranking above HIV/AIDS, TB was one of the leading causes of death from a single infectious agent in 2017 globally [1].

More than 130 years ago, after centuries of fatally scourging mankind, Robert Koch had finally and accurately identified and cultured the etiological agent of tuberculosis, named as tubercle bacillus on that time [2]. Originating from the family Mycobacteriaceae, TB is particularly caused by several strains of *Mycobacterium tuberculosis* complex, encompassing *Mycobacterium tuberculosis* itself, *M. africanum, M. bovis, M. caprae, M. microti, M. pinnipedii* and *M. cannetii* [3]. These strains of Mycobacteria have over 95% similarities at the nucleotide level but they have major differences in terms of hosts, phenotypes and pathogenesis [4]. While *M. bovis* is the specific causative tuberculosis agent of bovines, *M. tuberculosis* is on the other hand, exclusive to humans as its host [4]. One distinct characteristic of *M. tuberculosis* that set it apart from Gram positive and Gram negative bacteria is the presence of long fatty acid chains on its cell envelope, called as mycolic acids layer [5]. Being concertedly present with other “free” lipids and lipoglycans, the mycolic acids layer confers a surface on *Mycobacteria* that is impermeable to standard Gram staining and therapeutic agents [5,6,7]. Since their first discovery in the 1950s, TB-affected patients have since been relying on the current anti-TB drugs, which are divided into three main groups: First-line anti-TB drugs (isoniazid, rifampicin, pyrazinamide, ethambutol and streptomycin), second-line anti-TB drugs (kanamycin, capreomycin, amikacin and fluoroquinolones) and third-line anti-TB drugs (clofazimine, linezolid, amoxicillin/clavulanate, imipenem/cilastatin and clarithromycin) [8]. By possessing different drug targets which resulted in different antimycobacterial effects such as inhibiting mycolic acid synthesis, transcription, translation and arabinogalactan synthesis, the first-line TB drugs were proven efficacious for TB therapy, with more than 95% cure rates under ideal conditions of direct observation by the patients’ carriers [3]. Despite the high level of efficacy of aforementioned anti-TB drugs, they faced several challenges. The implementation of existing standard anti-TB regiment in hospitals is a lengthy treatment of at least six months. This in return has led to compliance issues within TB patients [3]. Moreover, the current anti-TB drugs were observed to cause adverse side effects [9]. Detrimental side effects ranging from the minor ones such as nausea, vomiting, fever and headache to the extent of major ones such as psychosis, hyperuricemia, ototoxicity and hepatotoxicity were exhibited from patients undergoing current anti-TB drug regimen [9,10,11]. The emergence of new strains of *M. tuberculosis*, multi-drug resistance tuberculosis (MDR-TB) and extensively-drug resistance tuberculosis (XDR-TB) has since exacerbated this global health problem [12,13]. Therefore, an effort of discovering a novel anti-TB drug should be commenced, as a sign of support to both Sustainable Development Goal 3 and End TB Strategy [1].

Owing to their diversity and abundance, medicinal plants offer new hope to physicists and scientists by serving as a sustainable source for novel leads in diseases therapy [14,15]. Plants have been used as primary medicinal and therapeutic sources by mankind since the earliest human civilizations [16]. Being affordable and easily accessible, plants are still being utilized for medicinal purposes particularly to treat TB until today, by people around the globe [17,18,19,20]. Plants are usable for medicinal purposes as a result of the presence of its wide array of secondary metabolites within them [21].

Plant secondary metabolites, also known as phytochemicals, are chemicals that are present naturally in plants. Unlike primary metabolites, these chemicals do not directly take part in the growth, reproduction and development of plants [22]. However, their absence may disrupt the plants survivability on a long-term basis [23]. Phytochemicals are also distinct from primary metabolites in a way that their distribution is limited in the plant kingdom [24]. In other words, particular phytochemicals are present only in a specific species or related species of plant [24]. Phytochemicals are basically classified based on chemical structure composition, their solubility in solvents and biosynthesis pathways [25]. Though they do not significantly involve in the crucial functions of plant survivability, phytochemicals are the key players that contribute the specific odors, colour and taste of plant parts [22]. As a sessile organism with lack of immunity system, plants rely chiefly on these secondary metabolites to defend themselves against both biotic and abiotic stresses [26]. Biological activities particularly antimycobacterial activity displayed by particular plants are actually closely-related with the phytochemicals contained within the plants as these chemicals are the ones responsible in characterizing such activity [27,28].

For the past 17 years, literature has shown several main review publications associated with the main groups of phytochemicals in plants relative to its pivotal role in antimycobacterial activity. Newton et al. [29] have reviewed and identified plant species, which exhibited antimycobacterial activity, also describing their extracts and active constituents involved. In a review conducted by Copp [30], natural products, from year 1990 to year 2012, with inhibitory activity against mycobacteria are widely covered. The review was arranged in chemical structural class to highlight possibilities of any structure-activity relationships. Another review pertaining to natural products possessing antimycobacterial capability was done by Okunade et al. [31]. In their article, 88 bioactive compounds of different classes from plants, marine organism, fungi and one bacterium that exhibited antimycobacterial characteristic at minimal inhibitory concentrations (MIC) of < 200 µg/mL were reviewed. In a review published by Gautam et al. [32], different species of plants species coming from numerous families were elucidated with respect to their parts and extracts used for bioassay against different strains of Mycobacteria. MIC of each plant species antimycobacterial activity was also included. A review by Rogoza et al. [33] described a vast array of both natural and synthetic products that possessed MIC of 5 µg/mL or less against mycobacteria, all of which covered publications from year 2001 to year 2007. In their review, Garcia et al. [34] have compiled 278 nature-based products and some derivatives from plants, algae, fungi, cyanobacteria and sponges that showed a promising antimycobacterial property at MIC of less than 5 µg/mL. The review covered literature publications from year 2006 to year 2011. Two years later, in 2014, Santhosh et al. [35] described 127 compounds of different classes originating from 58 species of plants, which demonstrated antimycobacterial activity.

Amongst the aforementioned eleven major review publications of nature-derived antimycobacterial compounds, none of them specifically and individually focused on exploring into the potentials of phenolic compounds as novel antimycobacterial agents for TB therapy. Whereas phenolic compounds were shown to have positive antimycobacterial activity in all of the stated review publications, therefore an updated, extensive review that further probe into their potentials as an anti-TB drug candidate is imperative.

The present review attempted to bridge the gap by firstly, describing bioassay guidance, a current method widely used in plant-based drug discovery programme and elaborating general characteristics of phenolic compounds. Secondly, this review then focuses on compiling and arranging anti-TB phenolic compounds from natural products for the past 17 years (2000–2017), according to their origins (from which they were isolated) isolated active phenolic constituents and MIC against Mycobacterial strains. Phenolic compounds presented in past review publications within these 17 years were also included in the present review. This review also attempted to describe the mechanism of actions (MOA) of phenolic compounds involved in strong MIC.

## 2. Bioassay Guidance: A Multidisciplinary Drug Discovery Program that Integrate Chemistry and Biology Techniques

Bioassay-guided fractionation is currently, the latest technique utilized by natural products researchers to screen and identify bioactive compound(s) in natural product crude extract(s). As it is a process that constitutes alternating steps of biological assay and chemical fractionation, it significantly aids researchers in screening and selecting only isolated compound(s) that show bioactive characteristics towards test organisms. For the past two decades, the rapid advancements of technology in chromatography and spectroscopy have made it possible for the sensitivity of chemical fractionation to be enhanced greatly. This in turn, has paved a new way for researchers to explore both unstudied materials and previously studied genera, which then grants access to novel bioactive compounds and chemical derivatives [36,37,38].

### 2.1. Test Organism

It is clear that the test organism ideally used for the anti-TB drug discovery attempt is the TB causative agent itself, *M. tuberculosis*. The prominent pathogenic strain *M. tuberculosis* H_37_R*v* (ATCC 27294) is the strain that adequately represents the majority of *M. tuberculosis* clinical isolates since its drug susceptibility profile closely resembles those of the clinical isolates. However, employing a pathogenic type of *M. tuberculosis* strain requires biosafety level 3 laboratory (BL-3), which needs extra precautionary and safety steps for researchers in handling it. Due to this reason, researches may turn to alternative non-pathogenic strains of *M. tuberculosis* such as *M. smegmatis* (ATCC 607), *M. bovis* (ATCC 35743), and *M. tuberculosis* H_37_R*a* (ATCC 25177). *M. smegmatis* is a rapidly-growing and saprophytic mycobacteria while on the other hand *M. bovis* and *M. tuberculosis* H_37_R*a* are slow-growing mycobacteria. However, the aforementioned slow-growing strains mycobacteria resembled much closer to the *M. tuberculosis* H_37_R*v* with regard to both drug susceptibility profile and genetic composition. Since these strains are non-pathogenic, handling them only requires biosafety level 2 laboratory [36,37].

### 2.2. In Vitro Bioassays for Anti-TB Activity Evaluation

#### 2.2.1. Agar Diffusion

Agar diffusion, either well or disk, is a conventional method used widely in antimicrobial bioassays of natural products. However, one of the main drawbacks of applying this method is that it is unable to quantify natural products crude or isolated bioactive compounds due to the fact that, the result yielded by this method only indicates the presence or absence of growth inhibition at an undetermined concentration along the concentration gradient [38]. In addition, the size of inhibition zone chiefly depends on both the rate of diffusion of bioactive compounds and rate of growth of test organism, which means that the size of zone of inhibitions can only be understood as indications to either microbial vulnerability or resistance to well-defined antibiotics. In the context of mycobacterial bioassays, the application of agar diffusion is much more irrelevant. As generally known, mycobacteria possess high lipid content in their cell envelope which confers them impermeable to polar compounds, while at the same time, are highly-susceptible to non-polar compounds. These compounds will diffuse notably slower than polar compounds of similar molecular weight on aqueous agar. This ultimately results in a smaller inhibition zone, which is frequently and mistakenly perceived as weak antimicrobial activity. Moreover, active polar compounds with low molecular weight may diffuse to the equilibrium state even before the formation and appearance of colonies of slow-growing microorganisms. Should the equilibrium concentration be below Minimum Inhibitory Concentration (MIC), inhibition zones will never appear [36,37].

#### 2.2.2. Micro/Macro Agar Dilution

In this method, researchers are able to adjust variations of concentrations of extracts, fractions or compounds in the agar medium according to their own preferences. This beneficially allows them to determine the MIC value and quantify its activity. Most mycobacterial strains such as *M. tuberculosis* are able to grow effectively on Middlebrook 7H10 or 7H11 agar supplemented with oleic acid, albumin, dextrose and catalase (OADC). The procedure involved in this method firstly begins with the addition of a sample to the semisolid media (held at 50 °C) at a final concentration of 1% *v*/*v*. Subsequently, researchers may opt to either add 100–200 µL to 96-well microplates, 1.5 mL to 24-well microplates, 4 mL to 6-well microplates or 20 mL into standard Petri dishes of 150 mm diameter. As the medium hardened, by using micropipette, the inoculum can then be spotted on the surface of the agar. Recommended volumes of inoculum are 1–5 µL for 96-well microplates, 10 µL for 6-24-well microplates and 100 µL for standard Petri dishes. The plates are then incubated at 37 °C overnight. With that being said, the plates should then be inverted for the remaining period of incubation. However, the main disadvantage of this method that should be taken into consideration by researchers is the lengthy period (minimum of 18 days) for the appearance of the colony of *Mycobacteria* [36,37,38].

#### 2.2.3. Micro Broth Dilution

Using this method in microplates with 96-wells as antimycobacterial bioassay of natural products offers advantages such as little sample requirement, cost-effective and high-throughput. In such method, *Mycobacterial* strains are commonly cultured in Middlebrook 7H9 broth supplemented with glycerol (0.5%), casitone (0.1%), Tween-80 (0.05%) and ADC (10%) [38]. The presence or absence of growth of test organism in the broth can be simply quantified by turbidity in the broth medium. However, problems such as aggregation in the bacterial culture and precipitation of crude extracts of the natural product itself may render the result inaccurate and unreliable. Nevertheless, inclusion of a surfactant, Tween-80 in the bioassay reagents will reduce the ability of mycobacterial cells to adhere to one another, hence increases the available surface area of mycobacterial cells. This essentially helps to reduce aggregation in bacterial culture thus enhancing the result accuracy of the bioassay [39]. In addition, correct selection of solvents for plants extraction based on polarity of targeted compounds and introducing pH differences may further improve separation of acid, neutral and basic constituents, which ultimately reduce precipitation [40]. Utilization oxidation/reduction indicator dye like Alamar blue may significantly increase the sensitivity and rapidness of this assay. Researchers may then observe the result yielded by the Microplate Alamar Blue Assay (MABA) by simply reading it visually or by instrumentations such as microplate spectrophotometer or microplate fluorometer. In addition, for non-fluorometric readouts, ones may also opt for tetrazolium dyes. Bioassays through microbroth dilution are therefore a currently reliable and relevant method for test organism involving *Mycobacteria*. Furthermore, these assays enable researchers to determine even partial inhibition, to which it can be achieved by determining the relative activity of fractions from crude extracts using different concentrations [34,35]. It is crucial, however, for researchers to take precautionary steps when performing bioassays using such dyes. Resazurin is photosensitive, therefore requires that the incubation be done in the dark [41]. There are, in many cases, test compounds that interact with the assay chemistry. Hence, negative and positive controls must be empirically determined to ensure that there are no non-specific interactions with assay chemistry, which would lead to artifacts or false-positive or negative results [41]. Recognizing the possible interference that an assay may be susceptible to and designing orthogonal assays to confirm compound activity are the best ways to identify artifactual activities early in the probe or drug discovery programme [42]. Information of several types of artifactual activities have been discussed in a study by Thorne et al. [42] to which, future researchers can refer, for a better and accurate result as yielded from dye-based bioassays.

### 2.3. Sample Extraction

Extraction of samples involved the use of various types of chemical solvents. The selection of what solvents to use in an extraction procedure is precisely dependent on the texture and water content of the plant material and on the type of compounds that is intended to be isolated [43]. Common solvents used in natural sample extraction are, arranged in increasing polarity, as follows: Hexane, petroleum ether, ethyl acetate, chloroform, ethanol and methanol. In the context of extracting phenolic compounds, semi-polar and polar solvents are required as phenolic compounds are water-soluble compounds [43]. In addition, it is generally known that organic extracts of medium polarity are likely to contain phytochemicals including terpenoids, polyphenols and alkaloids with bioactive activity [44]. Past researches have revealed that employment of polar solvents such as methanol and ethanol have successfully yielded extracts with phenolic compounds [44,45,46]. In addition, the use of the solvent with medium polarity such as chloroform was also a success in an attempt to obtain phenolic compounds [47]. Crude extracts obtained from this particular step may then proceed for in vitro anti-TB bioassay stated in Section 2.2.3 to screen out extract with no bioactivity towards *M. tuberculosis*. Thus, only the extract that shows bacterial growth inhibition will be selected to proceed with the next step, which is fractionation and isolation.

### 2.4. Crude Extracts Fractionation and Isolation

Bioactive crude extracts obtained from Section 2.3 is comprised of various, unspecified mix of compounds, of which, some of them may exhibit bioactivity while others may not. Therefore, the extract will then need to go to further separation and purification steps, also referred to as fractionation and isolation, to accurately specify the compound(s) that is responsible for the bioactivity. The purification of natural constituents mainly encompasses one or another, or a combination of several chromatographic techniques such as column chromatography (CC), thin-layer chromatography (TLC), gas chromatography (GC), liquid chromatography and gas liquid chromatography (GLC) [43]. Again, guided by the in vitro anti-TB bioassay mentioned in Section 2.2.3, fractions obtained from chromatography techniques undergone repeated fractionation and isolation through chromatography techniques to obtain sub-fractions, which are purer. This process continues until the final compound is obtained [44,47]. Final compound obtained through repeated fractionation is an isolated and pure bioactive compound, which then may proceed for chemical identification and characterization.

### 2.5. Isolated Compounds Identification and Characterization

The final step in the bio-guided fractionation is the identification and characterization of the pure, isolated compound obtained from Section 2.4. However, it is crucial to firstly determine the class of compound and which substance it is within that class. This can be achieved by employing the compound’s reaction towards colour tests, its solubility, *R_f_* properties and UV spectral characteristics [43]. Then, researchers may continue for complete identification of the particular substance by measuring its spectral characteristics. Few common instrumentations to measure spectral characteristics of a compound: Ultra-violet (UV), infra-red (IR), nuclear magnetic resonance (NMR) and mass spectral (MS). A bioassay-guided fractionation by Cunha et al. [48] has led to the discovery and isolation of cassic acid (a bioactive anthraquinone) in *Cassia bakeriana* Craib bark dichloromethane extract for the first time. In the research, Cunha et al. [48] has utilized several spectroscopic instrumentation namely Fourier-transform infrared spectroscopy (FTIR), ultraviolet-visible spectroscopy (UV-vis), liquid chromatography-electrospray ionization-tandem mass spectrometry (LC-ESI-MS/MS), proton NMR (H^1^ NMR), correlation spectroscopy (COSY) and heteronuclear single-quantum correlation (HSQC). In another research by Fomogne-Fodjo et al. [44], through bioassay-guided fractionation and the use of spectroscopic instrumentations such as H NMR, C NMR, distortionless enhancement by polarization transfer (DEPT) and heteronuclear multiple-bond correlation (HMBC), two novel phenolic compounds, tetracosanoate and N-hydroxy immidiate-tetracaine were discovered.

## 3. Phenolic Compounds: Promising Nature-Derived Anti-Tuberculosis Compound

### 3.1. Nature-Derived Phenolic Active Compounds against Mycobacterium

Being one of the numerous classes of phytochemicals, phenolic compounds are among the most diverse class of phytochemicals and widely spread across the plant kingdom, of which 8000 of their structures have been reported [23]. Being present in abundance across plants with vast types of structures, the main characteristic that all of these phenolic compounds shared in common is that these compounds consist of an aromatic phenyl ring attached with one or more hydroxyl substituents [43]. The common carbon skeleton building block for phenolic compounds is C_6_-C_3_ phenylpropanoid unit [49]. Phenolic compounds are all aromatic and their pigments can be visually coloured [43]. They are commonly found combined with sugars as glycosides, thus they are prone to be water-soluble [43]. On the basis of their structure, phenolic compounds can be further classified into two main groups; flavonoids (the largest group of phenolic compounds) and non-flavonoids. Phenolic compounds from numerous plant sources, with their names, MIC and corresponding references are compiled in Table 1.

### 3.2. Mechanism of Action of Phenolic Compounds against Mycobacterium

As mentioned earlier, the emergence of resistance strains of Mycobacteria, MDR-TB and XDR-TB, has aggravated this global health problem in the past recent years. MDR-TB is caused by M. tuberculosis that is generally resistant to first-line anti-TB drugs (isoniazid and rifampicin) while XDR-TB is due to the resistance of *M. tuberculosis* against first-line anti-TB drugs (isoniazid and rifampicin), any fluoroquinolones and at least one of three injectable second-line anti-TB drugs (kanamycin, capreomycin and amikacin) [76,77]. The resistance in Mycobacteria, is basically divided into two types, namely, acquired resistance and intrinsic resistance. The former is closely associated with mutations of chromosomes in the genes encoding drug targets or drug activating enzymes [50,78]. This results in the alteration of the structure of the target protein, hence reducing the susceptibility of the bacteria to a particular drug [79]. It is noteworthy to know that the intrinsic resistance of TB is largely a man-made phenomenon as it is caused by such factors as poor physician prescription, poor patient adherence, poor supply or quality of drugs and difference of metabolism and nutrition [80]. On the other hand, intrinsic resistance is mainly attributed to cell wall permeability and systems efflux pumps that mediates only selected solutes, be it hydrophilic or hydrophobic, to enter into the bacteria and extruding foreign compounds out from the bacteria, respectively [50,79,81]. This type of resistance ultimately reduces the efficacy of current drugs significantly by both, causing them unable to enter into the Mycobacteria to their protein targets and expelling them before they could even reach their protein targets [82]. In the following sections, we will discuss several selected examples of Mycobacteria intrinsic resistance associated with reported phenolic compounds that act against them.

#### 3.2.1. Mycobacteria Efflux System Inhibition

Efflux pumps in MDR, an intrinsic resistance in TB, are able to transport a wide range of chemically and structurally unrelated compounds, which also impair the efficacy of different antibiotics [50]. To find a novel nature-derived efflux inhibitor is therefore imperative in overcoming this problem and phenolic compounds proved to be a promising candidate as an efflux inhibitor.

In a research conducted by Groblacher et al. [50], four active compounds have been isolated from the hexane extract of *Alpinia katsumadai* Hayata seeds, of which the fourth compound was identified as flavanone pinocembrin (dihydrochrysin). The research revealed that all of the compounds exhibited weak antimycobacterial activity, however they demonstrated EtBr efflux inhibitory activity comparable to reference efflux-pump inhibitors (EPIs). Specifically, the flavanone showed EtBr efflux inhibitory activity comparable to carbonyl cyanide *m*-chlorophenylhydrazone (CCCP), one of the reference EPI. Another study done by Lechner et al. [78] has displayed several phenolic compounds that presented EtBr inhibitory activity, which includes biochanin A, luteolin and resveratrol. Among the stated phenolic compounds, biochanin A achieved inhibition levels comparable to the standard EPI.

#### 3.2.2. Mycobacteria Proteasome Inhibition

Proteasome in MTB plays a crucial role in providing intrinsic resistance against deleterious effects of reactive nitrogen intermediates (RNI) [82,83,84,85,86]. RNI such as nitric oxide (NO) and radical NO_2_ are produced by inducible nitric oxide synthase (iNOS) in activated macrophage, all of which inflict nitrosative stress to MTB [82,84]. NO may also combine with superoxide from bacterial 3metabolism to form peroxynitrite that inflict oxidative damage to MTB [82]. Nitrous acid, a protonated form of NO on the other hand, generates an acidic environment in the phagosome of activated macrophage that may impair the survival of MTB as well [83]. Study by Gandotra et al. [85] has revealed that the silencing of *prcA* and *prcB*, genes that serve as building blocks for proteasomal structure disrupts MTB ability to persist in both in vitro and in vivo growth. Proteasome, which is involved in the degradation of proteins that are irreversibly oxidized, nitrated or nitrosated are thus vital in protecting MTB against injuries inflicted by RNI [82,83,84]. The MTB proteasome may therefore be a plausible target for novel anti-TB natural products, particularly phenolic compounds may serve as a proteasome inhibitor candidate. Zheng et al. [86] has screened 100 natural products for their inhibitory activities against MTB proteasome and 22 of them were phenolic compounds; flavonoids, coumarins, phenols and lignans. The phenolic compounds have demonstrated IC_50_ values ranging from 3.05% to as high as 88.69%. Interestingly, 10 of the 22 phenolic compounds were flavonoids, which exhibited more than 65% inhibitory activity. The flavonoids include baicalein, pectolinarin, quercetin, hispidulin, myricetin, isoliquiritigenin, icariin, kaempferol, curcumin and baicalin. The study has also revealed the association between the functional group substitution of the A- and C-ring in the flavonoid structure (hydroxyl residues at the C_3_ of the C-ring and hydroxyl/methoxy residues at the C_6_ of the B-ring) and the magnitude of inhibitory activity of the particular flavonoid. The study conclusively stated that flavonoids, being one of the sub-classes in phenolic compounds, might serve as a potential proteasome inhibitor.

#### 3.2.3. Mycolic Acid Biosynthesis Inhibition

Mycolic acids are high-molecular-weight C_60_-C_90_ α-alkyl, β-hydroxy long chain fatty acids that serve as a unique component of the mycobacterial cell wall [5,6,87,88,89]. These mycolic acids added with “free” (noncovalently linked) lipids and lipogylcans that exist in the outer leaflets of the cell wall, formed a highly impermeable barrier that confer mycobacterial intrinsic resistance against common bacterial Gram-staining and hydrophilic anti-TB therapeutic agent [87]. Mycolic acids are also associated with the pathogenicity and ability of *Mycobacteria* to form biofilms [88]. Due to the crucial defensive role played by mycolic acids, its biosynthesis is thus implicated as a potential target for nature-derived anti-TB agents, specifically phenolic compounds.

A recent study by Baquero et al. [90] has revealed the antimycobacterial activity of five compounds, of which three of them were polyphenolic compounds, particularly lignans (dihydrocubebin, hinokinin, ethoxycubebin) while the remaining two were synthetic compounds. Of all three lignans, ethoxycubebin has demonstrated the highest inhibitory activity with MIC value of 24 µg/mLm thus was further analyzed in terms of its effect on mycolic acid biosynthesis and ultrastructure. Consequently, the study has shown that ethoxycubebin caused a remarkable reduction in all mycolic acids after 24-h of its treatment to MTB H_37_R*v*. In addition to that, the ultrastructural analysis of the bacteria treated with ethoxycubebin has clearly displayed aberrant morphology with irregular and disorganized shapes. The study then discussed the possible correlation between mycolic acid levels and the composition of the cell wall that determines the overall structure of bacterial morphology and conclusively stated that lignans possessed a promising potential as antimycobacterial agents that affect mycolic acid metabolism.

#### 3.2.4. Nitric Oxide Inhibition

Nitric oxide are produced enzymatically by three isoforms of nitric oxide synthases namely, nNOS- the neuronal form, iNOS- inducible nitric oxide synthase present in various cell types such as macrophage and eNOS- primarily present in endothelium [91]. Serving the purpose as a pro-inflammatory mediator, nitric oxide is generated by activated macrophages upon initiation by inflammatory cytokines such as IFN-γ, TNF-α or IL-1 as well as toxins by bacteria or mycobacteria such as lipopolysaccharide (LPS) [68,91,92]. Inflammatory response is intended to provide protection against invading pathogens, however over production of NO may lead to diseases such as arterial hypotension, vasoplegia, lactic acidosis and others [91].

Past study by Costa et al. [68] has isolated two two flavonoids namely isoquercetin and afzelin from the leaves of *Ocotea notata*. The compounds were then analysed for their antimycobacterial activity and were found to show no antimycobacterial activity. However, both of the compounds exhibited significant results in inhibiting NO production by macrophages, with *p* < 0.001 at concentrations of 0.8, 4, 20 and 100 µg/mL when compared with the positive result. In addition, the NO production inhibition capability of those flavonoids were analysed to be higher than their cytotoxicity. The study implicated that despite the negative antimycobacterial activity of both of the flavonoids, they on the other hand were capable of acting as anti-inflammatory agents which was evident from its NO production inhibition result. In another study by Bernardes et al. [73], a flavonoid apigenin was isolated from the methanolic fruit extract of *Schinus terebinthifolius* and evaluated for its NO production inhibition activity, antioxidant activity and antimycobacterial activity. The result from the study has revealed that the flavonoid showed antimycobacterial activity of 14.53 ± 1.25 µg/mL at IC_50_. Furthermore, the apigenin also showed a significant NO production inhibition at IC_50_, which the value was 19.23 ± 1.34 µg/mL. The study conclusively stated that apigenin worked well as both antimycobacterial and anti-inflammatory agents.

## 4. Concluding Remarks

Tuberculosis remains a global health problem, particularly in under-developed and developing countries. Despite systematic efforts and actions from the world organizations and governments, though shown a slight decrease in trend for last several years, TB continues to claim millions of lives around the globe. As pointed out in this review, the emergence of resistance strains of *Mycobacterium tuberculosis* and shortcomings of current clinically used TB chemotherapy are the factors contributing to current TB prevalence and occurrence.

Natural products, particularly phenolic compounds from plants, may serve as an unmatched natural reservoir of novel, chemically and structurally diverse compounds which hold a huge prospect of novel mechanism-of-actions against etiological agents of TB. In this review article, we have selected and compiled phenolic compounds with antimycobacterial activities with their plants of origins that were reported in previous studies for the past 17 years. The bioassay-guidance method and its sub-steps involved have been discussed. General description of Mycobacteria resistance and mechanism-of-actions of several phenolic compounds against Mycobacteria have also been discussed. In spite of significant anti-TB activity of numerous stated phenolic compounds, and in some cases, comparable to standard anti-TB drug regiments, none of them have been progressed further into clinical development. Many of the highly-potential phenolic compounds were also lack of cytotoxicity tests. It is critically recommended for anti-TB drug discovery researches to include cytotoxicity test in parallel to antimycobacterial tests to elucidate the selectivity of the compound of interest. Moreover, there is a need to initiate further efforts into the clinical development of promising phenolic compounds. The phenolic compounds covered in this review could provide potential leads for in vivo and clinical tests or could serve as templates for further medicinal chemistry programme.

## Figures and Tables

**Table 1:** Minimum inhibition concentrations of selected plant phenolic compounds against Mycobacteria.

Compound Name	Plant Source	MIC (µg/mL)	References
pinocembrin (**1**)	*Alpinia katsumadai* Hayata	128 ^c^	[50]
eupomatenoid-1 (**2**)	*Aristolochia elegans* Mast.	100 ^a^	[51]
fargesin (**3**)	*Aristolochia elegans* Mast.	50 ^a^	[51]
(8R,8′R,9R)-cubebin (**4**)	*Aristolochia elegans* Mast.	100 ^a^	[51]
flavonoid 7-demethylartonol E (**5**)	*Artocarpus rigidus* Blume	50 ^b^	[52]
chromone artorigidusin (**6**)	*Artocarpus rigidus* Blume	12.5 ^b^	[52]
artonol B (**7**)	*Artocarpus rigidus* Blume	100 ^b^	[52]
artonin F (**8**)	*Artocarpus rigidus* Blume	6.25 ^b^	[52]
cycloartobiloxanthone (**9**)	*Artocarpus rigidus* Blume	25 ^b^	[52]
artoindonesianin C (**10**)	*Artocarpus rigidus* Blume	12.5 ^b^	[52]
(-)-butin (**11**)	*Butea monosperma* (Lam.) Taub.	25 ^b^	[53]
butein (**12**)	*Butea monosperma* (Lam.) Taub.	12.5 ^b^	[53]
butrin (**13**)	*Butea monosperma* (Lam.) Taub.	50 ^b^	[53]
monospermoside (**14**)	*Butea monosperma* (Lam.) Taub.	25 ^b^	[53]
(+)-isomonospermoside (**15**)	*Butea monosperma* (Lam.) Taub.	25 ^b^	[53]
7,3′, 4′-trihydroxyflavone (**16**)	*Butea monosperma* (Lam.) Taub.	50 ^b^	[53]
dihydromonospermoside (**17**)	*Butea monosperma* (Lam.) Taub.	50 ^b^	[53]
isoliquiritigenin (**18**)	*Butea monosperma* (Lam.) Taub.	25 ^b^	[53]
(-)-liquiritigenin (**19**)	*Butea monosperma* (Lam.) Taub.	25 ^b^	[53]
formononetin (**20**)	*Butea monosperma* (Lam.) Taub.	50 ^b^	[53]
afrormosin (**21**)	*Butea monosperma* (Lam.) Taub.	25 ^b^	[53]
formononetin-7-*O*-*β*-D-glucopyranoside (**22**)	*Butea monosperma* (Lam.) Taub.	100 ^b^	[53]
5,7-dihydroxy-4′-methoxyflavanone (**23**)	*Chromolaena odorata* (L.) R.M.King & H.Rob.	175.8 mM ^b^	[54]
4′-hydroxy-5,6,7′-trimethoxyflavanone (**24**)	*Chromolaena odorata* (L.) R.M.King & H.Rob.	606.0 mM ^b^	[54]
acetin (**25**)	*Chromolaena odorata* (L.) R.M.King & H.Rob.	704.2 mM ^b^	[54]
luteolin (**26**)	*Chromolaena odorata* (L.) R.M.King & H.Rob.	699.3 mM ^b^	[54]
kaempferol-3,7-dimethyl ether (**27**)	*Cnidoscolus chayamansa* McVaugh	>50 ^a^	[55]
5-hydroxy-7,3′,4′-trimethoxy flavanone (**28**)	*Cnidoscolus chayamansa* McVaugh	>50 ^a^	[55]
5,7,3′-trihydroxy-4′,5′-(2′′′′,2′′′′-dimethylpyran)-8,2¢-di(3-methyl-2-butenyl)-(2S)-flavanone (**29**)	*Dendrolobium lanceolatum* (Dunn) Schindl.	6.3 ^b^	[56]
5,7,3′-trihydroxy-4¢-methoxy-8,2′-di(3-methyl-2-butenyl)-(2S)-flavanone (**30**)	*Dendrolobium lanceolatum* (Dunn) Schindl.	12.5 ^b^	[56]
7,3′,4′-trihydroxy-6-methoxy-8,2′-di(3-methyl-2-butenyl)-(2S)-flavan.(**31**)	*Dendrolobium lanceolatum* (Dunn) Schindl.	25 ^b^	[56]
4′-hydroxy-2″,2″dimethyl-pyranoflavan (**32**)	*Dendrolobium lanceolatum* (Dunn) Schindl.	25 ^b^	[56]
8,4′-dimethoxy-7-O-γ,-dimethylallylisoflavone (**33**)	*Derris indica* (Lam.) Bennet	100 ^b^	[57]
3,4-methylenedioxy-10-methoxy-7-oxo[2]benzopyrano[4,3-b]benzopyran (**34**)	*Derris indica* (Lam.) Bennet	6.25 ^b^	[57]
desmethoxy kanugin (**35**)	*Derris indica* (Lam.) Bennet	50 ^b^	[57]
lacheolatin B (**36**)	*Derris indica* (Lam.) Bennet	50 ^b^	[57]
pongachromene (**37**)	*Derris indica* (Lam.) Bennet	100 ^b^	[57]
3,7-dimethoxyflavone (**38**)	*Derris indica* (Lam.) Bennet	50 ^b^	[57]
pachycarin D (**39**)	*Derris indica* (Lam.) Bennet	200 ^b^	[57]
maackiain (**40**)	*Derris indica* (Lam.) Bennet	50 ^b^	[57]
medicarpin (**41**)	*Derris indica* (Lam.) Bennet	100 ^b^	[57]
karanjachromene (**42**)	*Derris indica* (Lam.) Bennet	12.5 ^b^	[57]
pinnatin (**43**)	*Derris indica* (Lam.) Bennet	12.5 ^b^	[57]
isobachalcone (**44**)	*Dorstenia barteri var. multiradiata* (Engl.) Hijman & C.C.Berg	2.44 ^a,c^	[58]
kanzanol C (**45**)	*Dorstenia barteri var. multiradiata* (Engl.) Hijman & C.C.Berg	9.76 ^a^, 19.53 ^c^	[58]
4-hydroxylonchocarpin (**46**)	*Dorstenia barteri var. multiradiata* (Engl.) Hijman & C.C.Berg	9.76 ^a,c^	[58]
stipulin (**47**)	*Dorstenia barteri var. multiradiata* (Engl.) Hijman & C.C.Berg	39.06 ^a,c^	[58]
amentoflavone (**48**)	*Dorstenia barteri var. multiradiata* (Engl.) Hijman & C.C.Berg	39.06 ^a,c^	[58]
khonkloginols A (**49**)	*Eriosema chinense* Vogel	25 ^b^	[59]
khonkloginols B (**50**)	*Eriosema chinense* Vogel	50 ^b^	[59]
khonkloginols F (**51**)	*Eriosema chinense* Vogel	50 ^b^	[59]
khonkloginols H (**52**)	*Eriosema chinense* Vogel	25 ^b^	[59]
lupinifolinol (**53**)	*Eriosema chinense* Vogel	25 ^b^	[59]
dehydrolupinifolinol (**54**)	*Eriosema chinense* Vogel	12.5 ^b^	[59]
flemichin D (**55**)	*Eriosema chinense* Vogel	12.5 ^b^	[59]
eriosemaone (**56**)	*Eriosema chinense* Vogel	12.5 ^b^	[59]
lupinifolin (**57**)	*Eriosema chinense* Vogel	12.5 ^b^	[59]
3-hydroxyxanthyletin (**58**)	*Ficus nervosa* B.Heyne ex Roth	16 ^a^	[60]
xanthyletin (**59**)	*Ficus nervosa* B.Heyne ex Roth	220 ^a^	[60]
umbelliferone (**60**)	*Ficus nervosa* B.Heyne ex Roth	150 ^a^	[60]
scopoletin (**61**)	*Ficus nervosa* B.Heyne ex Roth	≥110 ^a^	[60]
carpachromene (**62**)	*Ficus nervosa* B.Heyne ex Roth	110 ^a^	[60]
genistein (**63**)	*Ficus nervosa* B.Heyne ex Roth	35 ^a^	[60]
prunetin (**64**)	*Ficus nervosa* B.Heyne ex Roth	30 ^a^	[60]
cajanin (**65**)	*Ficus nervosa* B.Heyne ex Roth	110 ^a^	[60]
apigenin (**66**)	*Ficus nervosa* B.Heyne ex Roth	70^a^	[60]
naringenin (**67**)	*Ficus nervosa* B.Heyne ex Roth	≤ 2.8 ^a^	[60]
(2*S*)-5,7,2′-trihydroxyflavone (**68**)	*Galenia africana* L.	110.20 µM ^c^, 367.60 µM ^a^	[61]
(*E*)—2′,4′-dihydroxychalcone (**69**)	*Galenia africana* L.	468.70 µM ^c^, 195.30 µM ^a^	[61]
(*E*)-3,2′,4′-trihydroxy-3′-methoxychalcone (**70**)	*Galenia africana* L.	174.80 µM ^a^	[61]
isoliquiritigenin (**71**)	*Glycyrrhiza glabra* L.	25 ^a^	[62]
liquiritigenin (**72**)	*Glycyrrhiza glabra* L.	26 ^a^	[62]
5-hydroxy-3,7,49-trimethoxyflavone (**73**)	*Haplopappus sonoriensis* (A.Gray)S.F.Blake	33% inhibition at 100 ^a^	[63]
5,7-dihydroxy-3,49-dimethoxyflavone (**74**)	*Haplopappus sonorensis* (A.Gray) S.F.Blake	98% inhibition at concentration of 100 ^a^	[63]
5,49-dihydroxy-3,7-dimethoxyflavone (**75**)	*Haplopappus sonorensis* (A.Gray) S.F.Blake	48% inhibition at concentration of 100 ^a^	[63]
3-cinnamoyltribuloside (**76**)	*Heritiera littoralis* Aiton	1600 ^d^, 800 ^e^	[64]
afzelin (**77**)	*Heritiera littoralis* Aiton	1600 ^e^	[64]
astilbin (**78**)	*Heritiera littoralis* Aiton	1600 ^d,e^	[64]
linaroside (**79**)	*Lantana camara* L.	30% inhibition of 6.25 ^a^	[65]
lantanoside (**80**)	*Lantana camara* L.	37%, inhibition 6.25 ^a^	[65]
dihydroguaiaretic acid (**81**)	*Larrea tridentate* (Sessé & Moc. ex DC.) Coville	50 ^a^	[66]
4-*epi*-larreatricin (**82**)	*Larrea tridentate* (Sessé & Moc. ex DC.) Coville	50 ^a^	[66]
3′-demethoxy-6-*O*-demethylisoguaiacin (**83**)	*Larrea tridentate* (Sessé & Moc. ex DC.) Coville	>50 ^a^	[66]
5,4′-dihydroxy-3,7,8,3′-tetramethoxyflavone (**84**)	*Larrea tridentate* (Sessé & Moc. ex DC.) Coville	>50 ^a^	[66]
5,4′-dihydroxy-3,7,8-trimethoxyflavone (**85**)	*Larrea tridentate* (Sessé & Moc. ex DC.) Coville	>50 ^a^	[66]
nevadensin (**86**)	*Limnophila geoffrayi* Bon.	200 ^b^	[67]
isothymusin (**87**)	*Limnophila geoffrayi* Bon.	200 ^b^	[67]
isoquercitrin (**88**)	*Ocotea notate* (Nees & Mart.) Mez	0	[68]
afzelin (**89**)	*Ocotea notate* (Nees & Mart.) Mez	0	[68]
7-methylquercetagetin (**90**)	*Paepalanthus latipes* Silveira	500 ^a^, 1000 ^g^	[69]
7-methylquercetagetin-4′-*O*-β-D-glucopyranoside (**91**)	*Paepalanthus latipes* Silveira	500 ^a^, 1000 ^g^	[69]
bakuchiol (**92**)	*Psoralea corylifolia* L.	15.79 ^f^ 21.4 ^h^ >500 ^c^	[70]
chrysophanol (**93**)	*Rheum rhaponticum* L.	64 ^b^ 128 ^h^	[71]
aloe-emodin (**94**)	*Rheum rhaponticum* L.	64 ^b^ 128 ^h^	[71]
rhaponticin (**95**)	*Rheum rhaponticum* L.	128 ^b^ 128 ^h^	[71]
resveratrol (**96**)	*Rheum rhaponticum* L.	128 ^b^ 64 ^h^	[71]
barbaloin (**97**)	*Rheum rhaponticum* L.	32 ^b^ 128 ^h^	[71]
deoxyrhaponticin (**98**)	*Rheum rhaponticum* L.	256 ^b^ 128 ^h^	[71]
precatorin A (**99**)	*Rhynchosia precatoria* (Willd.) DC	62.5 ^a^	[72]
precatorin B (**100**)	*Rhynchosia precatoria* (Willd.) DC	62.5 ^a^	[72]
precatorin C (**101**)	*Rhynchosia precatoria* (Willd.) DC	62.5 ^a^	[72]
lupinifolin (**102**)	*Rhynchosia precatoria* (Willd.) DC	31.25 ^a^	[72]
cajanone (**103**)	*Rhynchosia precatoria* (Willd.) DC	62.5 ^a^	[72]
apigenin (**104**)	*Schinus terebinthifolius* Raddi	14.5 ^h^	[73]
tetraceranoate (**105**)	*Tetracera potatoria* Afzel. ex G.Don	7.8 ^c^, 125 ^f^	[74]
N-hydroxy imidate-tetracerane (**106**)	*Tetracera potatoria* Afzel. ex G.Don	31 ^c^, 125 ^f^	[74]
(+)-1-hydroxy-2,6-bis-*epi*-pinoresinol (**107**)	*Valeriana laxiflora* DC.	127 ^a^	[75]
5,7-dihydroxy-3,6,4′-trimethoxyflavone (**108**)	*Valeriana laxiflora* DC.	46.2 ^a^	[75]
ferulic acid (**109**)	*Valeriana laxiflora* DC.	>128 ^a^	[75]
(+)-1-hydroxypinoresinol (**110**)	*Valeriana laxiflora* DC.	>128 ^a^	[75]
prinsepiol (**111**)	*Valeriana laxiflora* DC.	>128 ^a^	[75]
5,7,3′-trihydroxy-4′-methoxyflavone (**112**)	*Valeriana laxiflora* DC.	>128 ^a^	[75]

^a^ Antimycobacterial assay using *Mycobacterium tuberculosis H37Rv*; ^b^ Antimycobacterial assay using *M. tuberculosis H37Ra*; ^c^ Antimycobacterial assay using *M. smegmatis*; ^d^ Antimycobacterial assay using *M. madagascariense*; ^e^ Antimycobacterial assay using *M. indicus pranii*; ^f^ Antimycobacterial assay using *M. aurum*; ^g^ Antimycobacterial assay using *M. avium*; ^h^ Antimycobacterial assay using *M. bovis*.

## References

[1] (2018). WHO Global Tuberculosis Report 2018.

[2] Barnes D.S. (2000). Historical perspectives on the etiology of tuberculosis. Microbes Infect..

[3] Zumla A., Nahid P., Cole S.T. (2013). Advances in the development of new tuberculosis drugs and treatment regimens. Nat. Rev. Drug Discov..

[4] Jordao L., Vieira O.V. (2011). Tuberculosis: New aspects of an old disease. Int. J. Cell Biol..

[5] Jackson M. (2014). The mycobacterial cell envelope-lipids. Cold Spring Harb. Perspect. Med..

[6] Brennan P.J., Nikaido H. (1995). The Envelope of Mycobacteria. Annu. Rev. Biochem..

[7] Hett E.C., Rubin E.J. (2008). Bacterial growth and cell division: a mycobacterial perspective. Microbiol. Mol. Biol. Rev..

[8] Keshavjee S., Farmer P.E. (2012). Tuberculosis, drug resistance, and the history of modern medicine. N. Engl. J. Med..

[9] Zumla A., Raviglione M., Hafner R., von Reyn C.F. (2013). Tuberculosis. N. Engl. J. Med..

[10] Gulbay B.E., Gurkan O.U., Yildiz O.A., Onen Z.P., Erkekol F.O., Baccioglu A., Acican T. (2006). Side effects due to primary antituberculosis drugs during the initial phase of therapy in 1149 hospitalized patients for tuberculosis. Respir. Med..

[11] Arbex M.A., Varella M.D.C.L., Siqueira H.R.D., Mello F.A.F.D. (2010). Antituberculosis drugs: Drug interactions, adverse effects and use in special situations. Part 1: First-line drugs. Braz. J. Pulmonol..

[12] Tabarsi P., Mardani M. (2012). Extensively Drug-Resistant Tuberculosis: A Review Article. Arch. Clin. Infect. Dis..

[13] Gupta V.K., Shukla C., Bisht G.R., Saikia D., Kumar S., Thakur R.L. (2011). Detection of anti-tuberculosis activity in some folklore plants by radiometric BACTEC assay. Lett. Appl. Microbiol..

[14] Cragg G.M., Newman D.J. (2005). Biodiversity: A continuing source of novel drug leads. Pure Appl. Chem..

[15] Pathak K., Das R.J. (2013). Herbal Medicine- A Rational Approach in Health Care System. Int. J. Herb. Med..

[16] Hosseinzadeh S., Jafarikukhdan A., Hosseini A., Armand R. (2015). The Application of Medicinal Plants in Traditional and Modern Medicine: A Review of *Thymus vulgaris*. Int. J. Clin. Med..

[17] Nguta J.M., Appiah-Opong R., Nyarko A.K., Yeboah-Manu D., Addo P.G. (2015). Current perspectives in drug discovery against tuberculosis from natural products. Int. J. Mycobacteriol..

[18] Jimenez-Arellanes M.A., Gutierrez-Rebolledo G., Rojas-Tome S., Meckes-Fischer M. (2014). Medicinal Plants, an Important Reserve of Antimycobacterial and Antitubercular Drugs: An Update. J. Infect. Dis. Ther..

[19] Bunalema L., Obakiro S., Tabuti J.R., Waako P. (2014). Knowledge on plants used traditionally in the treatment of tuberculosis in Uganda. J. Ethnopharmacol..

[20] Akintola A.O., Kehinde A.O., Adebiyi O.E., Ademowo O.G. (2013). Anti-tuberculosis activities of the crude methanolic extract and purified fractions of the bulb of *Crinum jagus*. Niger. J. Physiol. Sci..

[21] Sharma D., Yadav J. (2016). An Overview of Phytotherapeutic Approaches for the Treatment of Tuberculosis. Mini-Rev. Med. Chem..

[22] Ahmed E., Arshad M., Khan M.Z., Amjad M.S., Sadaf H.M., Riaz I., Sabir S., Ahmad N., Sabaoon M.A. (2017). Secondary metabolites and their multidimensional prospective in plant. J. Pharmacogn. Phytochem..

[23] Irchhaiya R., Kumar A., Yadav A., Gupta N., Kumar S., Gupta N., Kumar S., Yadav V., Prakash A., Gurjar H. (2015). Metabolites in Plants and its Classification. World J. Pharm. Pharm. Sci..

[24] Anulika N.P., Ignatius E.O., Raymond E.S., Osasere O.-I., Abiola A.H. (2016). The Chemistry of Natural Product: Plant Secondary Metabolites. Int. J. Technol. Enhanc. Emerg. Eng. Res..

[25] Tiwari R., Rana C.S. (2015). Plant secondary metabolites: A review. Int. J. Eng. Res. Gen. Sci..

[26] Mazid M., Khan T., Mohammad F. (2011). Role of Secondary Metabolites in Defense Mechanisms of Plants. Biol. Med..

[27] Compean K.L., Ynalvez R.A. (2014). Antimicrobial Activity of Plant Secondary Metabolites: A Review. Res. J. Med. Plant.

[28] Kumar N., Banik A., Sharma P.K. (2010). Use of Secondary Metabolite in Tuberculosis: A Review. Der. Pharm. Chem..

[29] Newton S.M., Lau C., Wright C.W. (2000). A Review of Antimycobacterial Natural Products. Phytother. Res..

[30] Copp B.R. (2003). Antimycobacterial natural products. Natl. Prod. Rep..

[31] Okunade A.L., Elvin-Lewis M.P., Lewis W.H. (2004). Natural antimycobacterial metabolites: Current status. Phytochemistry.

[32] Gautam R., Saklani A., Jachak S.M. (2007). Indian medicinal plants as a source of antimycobacterial agents. J. Ethnopharmacol..

[33] Rogoza L.N., Salakhutdinov N.F., Tolstikov G.A. (2009). Natural and Synthetic Compounds with an Antimycobacterial Activity. Mini-Rev. Org. Chem..

[34] Garcia A., Bocanegra-Garcia V., Palma-Nicolas J.P., Rivera G. (2012). Recent advances in antitubercular natural products. Eur. J. Med. Chem..

[35] Santhosh R.S., Suriyanarayanan B. (2014). Plants: A source for new antimycobacterial drugs. Planta Med..

[36] Pauli G.F., Case R.J., Inui T., Wang Y., Cho S., Fischer N.H., Franzblau S.G. (2005). New perspectives on natural products in TB drug research. Life Sci..

[37] Nguta J.M., Appiah-Opong R., Nyarko A.K., Yeboah-Manu D., Addo P.G. (2015). Medicinal plants used to treat TB in Ghana. Int. J. Mycobacteriol..

[38] Sanusi S.B., Abu Bakar M.F., Mohamed M., Sabran S.F., Mainasara M.M. (2017). Southeast Asian Medicinal Plants as a Potential Source of Antituberculosis Agent. Evid. Based Complement. Altern. Med..

[39] O’Neill T.E., Li H., Colquhoun C.D., Johnson J.A., Webster D., Gray C.A. (2014). Optimisation of the microplate resazurin assay for screening and bioassay-guided fractionation of phytochemical extracts against *Mycobacterium tuberculosis*. Phytochem. Anal..

[40] Cos P., Vlietinck A.J., Berghe D.V., Maes L. (2006). Anti-infective potential of natural products: How to develop a stronger in vitro ‘proof-of-concept’. J. Ethnopharmacol..

[41] Rampersad S.N. (2012). Multiple applications of Alamar Blue as an indicator of metabolic function and cellular health in cell viability bioassays. Sensors.

[42] Thorne N., Auld D.S., Inglese J. (2010). Apparent activity in high-throughput screening: Origins of compound-dependent assay interference. Curr. Opin. Chem. Biol..

[43] Harbone J.B. (1973). Phytochemical Methods A Guide to Modern Techniques of Plant Analysis.

[44] Fomogne-Fodjo M.C., Van Vuuren S., Ndinteh D.T., Krause R.W., Olivier D.K. (2014). Antibacterial activities of plants from Central Africa used traditionally by the Bakola pygmies for treating respiratory and tuberculosis-related symptoms. J. Ethnopharmacol..

[45] Cardoso C.A.L., Coelho R.G., Honda N.K., Pott A., Pavan F.R., Leite C.Q.F. (2013). Phenolic compounds and antioxidant, antimicrobial and antimycobacterial activities of *Serjania erecta* Radlk. (Sapindaceae). Braz. J. Pharm. Sci..

[46] Askun T., Tekwu E.M., Satil F., Modanlioglu S., Aydeniz H. (2013). Preliminary antimycobacterial study on selected Turkish (Lamiaceae) against *Mycobacterium tuberculosis* and search for some phenolic constituents. BMC Complement. Altern. Med..

[47] Guan Y.F., Song X., Qiu M.H., Luo S.H., Wang B.J., Van Hung N., Cuong N.M., Soejarto D.D., Fong H.H., Franzblau S.G. (2016). Bioassay-Guided Isolation and Structural Modification of the Anti-TB Resorcinols from *Ardisia gigantifolia*. Chem. Biol. Drug Design.

[48] Cunha L.C.S., de Morais S.A.L., de Aquino F.J.T., Chang R., de Oliveira A., Martins M.M., Martins C.H.G., Sousa L.C.F., Barros T.T., de Silva C.V. (2017). Bioassay-guided fractionation and antimicrobial and cytotoxic activities of *Cassia bakeriana* extracts. Rev. Bras. Farmacogn..

[49] Pereira D.M., Valentão P., Pereira J.A., Andrade P.B. (2009). Phenolics: From Chemistry to Biology. Molecules.

[50] Groblacher B., Kunert O., Bucar F. (2012). Compounds of *Alpinia katsumadai* as potential efflux inhibitors in *Mycobacterium smegmatis*. Bioorg. Med. Chem..

[51] Jimenez-Arellanes A., Leon-Diaz R., Meckes M., Tapia A., Molina-Salinas G.M., Luna-Herrera J., Yepez-Mulia L. (2013). Antiprotozoal and Antimycobacterial Activities of Pure Compounds from *Aristolochia elegans* Rhizomes. Evid. Based Complement. Altern. Med..

[52] Namdaung U., Aroonrerk N., Suksamrarn S., Danwisetkanjana K., Saenboonrueng J., Arjchomphu W., Suksamrarn A. (2006). Bioactive Constituents of the Root Bark of *Artocarpus rigidus subsp. rigidus*. Chem. Pharm. Bull..

[53] Chokchaisiri R., Suaisom C., Sriphota S., Chindaduang A., Chuprajob T., Suksamrarn A. (2009). Bioactive Flavonoids of the Flowers of *Butea monosperma*. Chem. Pharm. Bull..

[54] Suksamrarn A., Chotipong A., Suavansri T., Boongird S., Timsuksai P., Vimuttipong S., Chuaynugul A. (2004). Antimycobacterial activity and cytotoxicity of flavonoids from the flowers of *Chromolaena odorata*. Arch. Pharm. Res..

[55] Perez-Gonzalez M.Z., Gutierrez-Rebolledo G.A., Yepez-Mulia L., Rojas-Tome I.S., Luna-Herrera J., Jimenez-Arellanes M.A. (2017). Antiprotozoal, antimycobacterial, and anti-inflammatory evaluation of *Cnidoscolus chayamansa* (Mc Vaugh) extract and the isolated compounds. Biomed. Pharmacother. Biomed. Pharmacother..

[56] Kanokmedhakul S., Kanokmedhakul K., Nambuddee K., Kongsaeree P. (2004). New Bioactive Prenylflavonoids and Dibenzocycloheptene Derivative from Roots of *Dendrolobium lanceolatum*. J. Nat. Prod..

[57] Koysomboon S., van Altena I., Kato S., Chantrapromma K. (2006). Antimycobacterial flavonoids from *Derris indica*. Phytochemistry.

[58] Kuete V., Ngameni B., Mbaveng A.T., Ngadjui B., Meyer J.J., Lall N. (2010). Evaluation of flavonoids from *Dorstenia barteri* for their antimycobacterial, antigonorrheal and anti-reverse transcriptase activities. Acta Trop..

[59] Suthhivaiyakit S., Thongnak O., Lhinhatrakool T., Yodchun O., Srimark R., Dowtaisong P., Chuankamnerdkarn M. (2009). Cytotoxic and Antimycobacterial Prenylated Flavonoids from the Roots of *Eriosema chinense*. J. Nat. Prod..

[60] Chen L.-W., Cheng M.-J., Peng C.-F., Chen I.-S. (2010). Secondary Metabolites and Antimycobacterial Activities from the Roots of *Ficus nervosa*. Chem. Biodivers..

[61] Mativandlela S.P.N., Muthivhi T., Kikuchi H., Oshima Y., Hamilton C., Hussein A.A., van der Walt M.L., Houghton P.J., Lall N. (2009). Antimycobacterial Flavonboids from the Leaf Extract of *Galenia africana*. J. Nat. Prod..

[62] Gaur R., Thakur J.P., Yadav D.K., Kapkoti D.S., Verma R.K., Gupta N., Khan F., Saikia D., Bhakuni R.S. (2015). Synthesis, antitubercular activity, and molecular modeling studies of analogues of isoliquiritigenin and liquiritigenin, bioactive components from *Glycyrrhiza glabra*. Med. Chem. Res..

[63] Murillo J.I., Encarnación-Dimayuga R., Malmstrøm J., Christophersen C., Franzblau S.G. (2003). Antimycobacterial flavones from *Haplopappus sonorensis*. Fitoterapia.

[64] Christopher R., Nyandoro S.S., Chacha M., de Koning C.B. (2014). A new cinnamoylglycoflavonoid, antimycobacterial and antioxidant constituents from *Heritiera littoralis* leaf extracts. Nat. Prod. Res..

[65] Begum S., Wahab A., Siddiqui B.S. (2008). Antimycobacterial activity of flavonoids from Lantana camara Linn. Nat. Prod. Res..

[66] Favela-Hernandez J.M., Garcia A., Garza-Gonzalez E., Rivas-Galindo V.M., Camacho-Corona M.R. (2012). Antibacterial and antimycobacterial lignans and flavonoids from *Larrea tridentata*. Phytother. Res..

[67] Suksamram A., Poomsing P., Aroonrerk N., Punjanon T., Suksamrn S., Kongkun S. (2003). Antimycobacterial and Antioxidant Flavones from *Limnophila geoffrayi*. Arch. Pharm. Res..

[68] Costa I.F., Calixto S.D., Heggdorne de Araujo M., Konno T.U., Tinoco L.W., Guimaraes D.O., Lasunskaia E.B., Leal I.R., Muzitano M.F. (2015). Antimycobacterial and nitric oxide production inhibitory activities of *Ocotea notata* from Brazilian restinga. Sci. World J..

[69] Moreira R.R.D., Martins G.Z., Pietro R.C.L.R., Sato D.N., Pavan F.R., Leite S.R.A., Vilegas W., Leite C.Q.F. (2013). *Paepalanthus* spp: Antimycobacterial activity of extracts, methoxylated flavonoids and naphthopyranone fractions. Rev. Bras. Farmacogn..

[70] Newton S.M., Lau C., Gurcha S.S., Besra G.S., Wright C.W. (2002). The evaluation of forty-three plant species for in vitro antimycobacterial activities; isolation of active constituents from *Psoralea corylifolia* and *Sanguinaria canadensis*. J. Ethnopharmacol..

[71] Smolarz H.D., Swatko-Ossor M., Ginalska G., Medyńska E. (2013). Antimycobacterial Effect of Extract and Its Components from *Rheum rhaponticum*. J. AOAC Int..

[72] Coronado-Aceves E.W., Gigliarelli G., Garibay-Escobar A., Zepeda R.E.R., Curini M., Lopez Cervantes J., Ines Espitia-Pinzon C.I., Superchi S., Vergura S., Marcotullio M.C. (2017). New Isoflavonoids from the extract of *Rhynchosia precatoria* (Humb. & Bonpl. ex Willd.) DC. and their antimycobacterial activity. J. Ethnopharmacol..

[73] Bernardes N.R., Heggdorne-Araújo M., Borges I.F.J.C., Almeida F.M., Amaral E.P., Lasunskaia E.B., Muzitano M.F., Oliveira D.B. (2014). Nitric oxide production, inhibitory, antioxidant and antimycobacterial activities of the fruits extract and flavonoid content of *Schinus terebinthifolius*. Rev. Bras. Farmacogn..

[74] Fomogne-Fodjo M.C., Ndinteh D.T., Olivier D.K., Kempgens P., van Vuuren S., Krause R.W. (2017). Secondary metabolites from *Tetracera potatoria* stem bark with anti-mycobacterial activity. J. Ethnopharmacol..

[75] Gu J.-Q., Wang Y., Franzblau S.G., Montenegro G., Yang D., Timmermann B.N. (2004). Antitubercular Constituents of *Valeriana laxiflora*. Planta Med..

[76] Gandhi N.R., Nunn P., Dheda K., Schaaf H.S., Zignol M., van Soolingen D., Jensen P., Bayona J. (2010). Multidrug-resistant and extensively drug-resistant tuberculosis: A threat to global control of tuberculosis. Lancet.

[77] Zumla A.I., Gillespie S.H., Hoelscher M., Philips P.P.J., Cole S.T., Abubakar I., McHugh T.D., Schito M., Maeurer M., Nunn A.J. (2014). New antituberculosis drugs, regimens, and adjunct therapies: Needs, advances, and future prospects. Lancet Infect. Dis..

[78] Lechner D., Gibbons S., Bucar F. (2008). Plant phenolic compounds as ethidium bromide efflux inhibitors in *Mycobacterium smegmatis*. J. Antimicrob. Chemother..

[79] Louw G.E., Warren R.M., Gey van Pittius N.C., McEvoy C.R., Van Helden P.D., Victor T.C. (2009). A balancing act: Efflux/influx in mycobacterial drug resistance. Antimicrob. Agents Chemother..

[80] Islam M.M., Hameed H.M.A., Mugweru J., Chhotaray C., Wang C., Tan Y., Liu J., Li X., Tan S., Ojima I. (2017). Drug resistance mechanisms and novel drug targets for tuberculosis therapy. J. Genet. Genom..

[81] Rodrigues L., Ramos J., Couto I., Amaral L., Viveiros M. (2011). Ethidiym bromide transport across *Mycobacterium smegmatis* cell wall: Correlation with antibiotic resistance. BMC Microbiol..

[82] Darwin K.H., Ehrt S., Gutierrez-Ramos J.-C., Weich N., Nathan C.F. (2003). The Proteasome of *Mycobacterium tuberculosis* Is Required for Resistance to Nitric Oxide. Science.

[83] Lin G., Li D., de Carvalho L.P., Deng H., Tao H., Vogt G., Wu K., Schneider J., Chidawanyika T., Warren J.D. (2009). Inhibitors selective for mycobacterial versus human proteasomes. Nature.

[84] Cerda-Maira F., Darwin K.H. (2009). The *Mycobacterium tuberculosis* proteasome: More than just a barrel-shaped protease. Microb. Infect..

[85] Gandotra S., Lebron M.B., Ehrt S. (2010). The *Mycobacterium tuberculosis* proteasome active site threonine is essential for persistence yet dispensable for replication and resistance to nitric oxide. PLoS Pathog..

[86] Zheng Y., Jiang X., Gao F., Song J., Sun J., Wang L., Sun X., Lu Z., Zhang H. (2014). Identification of plant-derived natural products as potential inhibitors of the *Mycobacterium tuberculosis* proteasome. BMC Complement. Altern. Med..

[87] Bhatt A., Molle V., Besra G.S., Jacobs W.R., Kremer L. (2007). The *Mycobacterium tuberculosis* FAS-II condensing enzymes: Their role in mycolic acid biosynthesis, acid-fastness, pathogenesis and in future drug development. Mol. Microbiol..

[88] Pawelczyk J., Kremer L. (2014). The Molecular Genetics of Mycolic Acid Biosynthesis. Microbiol. Spectr..

[89] North E.J., Jackson M., Lee R.E. (2014). New Approaches to Target the Mycolic Acid Biosynthesis Pathway for the Development of Tuberculosis Therapeutics. Curr. Pharm. Design.

[90] Baquero E., Quinones W., Ribon W., Caldas M.L., Sarmiento L., Echeverri F. (2011). Effect of an Oxadiaxoline and a Lignan on Mycolic Acid Biosynthesis and Ultrastructural Changes of *Mycobacterium tuberculosis*. Tuberculosis Res. Treat..

[91] Guzik T.J., Korbut R., Adamek-Guzik T. (2003). Nitric Oxide and Superoxide in Inflammation and Immune Regulation. J. Physiol. Pharmacol..

[92] Shen S.-C., Lee W.-R., Lin H.-Y., Huang H.-C., Ko C.-H., Yang L.-L., Chen Y.-C. (2002). In vitro and in vivo inhibitory activities of rutin, wogonin and quercetin on lipopolysaccharide-induced nitric oxide and prostaglandin E2 production. Eur. J. Pharmacol..

